# Computed tomography body composition and clinical outcomes following lung transplantation in cystic fibrosis

**DOI:** 10.1186/s12890-023-02398-4

**Published:** 2023-03-30

**Authors:** Ann L Jennerich, Lois Downey, Christopher H Goss, Siddhartha G Kapnadak, Joseph B Pryor, Kathleen J Ramos

**Affiliations:** 1 Department of Medicine, Division of Pulmonary, Critical Care and Sleep Medicine, University of Washington, Seattle, WA USA; 2 Department of Pediatrics, Division of Pulmonary and Sleep Medicine, University of Washington, Seattle, WA USA; 3 Department of General Internal Medicine, University of Washington, Seattle, WA USA

**Keywords:** Lung diseases, Lung transplantation, Nutritional status, Survival analysis

## Abstract

**Background:**

Low muscle mass is common in patients approaching lung transplantation and may be linked to worse post-transplant outcomes. Existing studies assessing muscle mass and post-transplant outcomes include few patients with cystic fibrosis (CF).

**Methods:**

Between May 1993 and December 2018, 152 adults with CF received lung transplants at our institution. Of these, 83 met inclusion criteria and had usable computed tomography (CT) scans. Using Cox proportional hazards regression, we evaluated the association between pre-transplant thoracic skeletal muscle index (SMI) and our primary outcome of death after lung transplantation. Secondary outcomes, including days to post-transplant extubation and post-transplant hospital and intensive care unit (ICU) length of stay, were assessed using linear regression. We also examined associations between thoracic SMI and pre-transplant pulmonary function and 6-min walk distance.

**Results:**

Median thoracic SMI was 26.95 cm^2^/m^2^ (IQR 23.97, 31.32) for men and 22.83 cm^2^/m^2^ (IQR 21.27, 26.92) for women. There was no association between pre-transplant thoracic SMI and death after transplant (HR 1.03; 95% CI 0.95, 1.11), days to post-transplant extubation, or post-transplant hospital or ICU length of stay. There was an association between pre-transplant thoracic SMI and pre-transplant FEV1% predicted (b = 0.39; 95% CI 0.14, 0.63), with higher SMI associated with higher FEV1% predicted.

**Conclusions:**

Skeletal muscle index was low for men and women. We did not identify a significant relationship between pre-transplant thoracic SMI and post-transplant outcomes. There was an association between thoracic SMI and pre-transplant pulmonary function, confirming the potential value of sarcopenia as a marker of disease severity.

**Supplementary Information:**

The online version contains supplementary material available at 10.1186/s12890-023-02398-4.

## Introduction

Cystic fibrosis (CF) is associated with significant pulmonary disease, and progressive respiratory failure is a common cause of death [1, 2]. Outcomes have improved significantly, but lung transplantation remains an important treatment option that can improve survival and quality of life for many people with advanced CF lung disease [3–5]. Selecting optimal candidates for lung transplantation includes evaluating risk factors for adverse post-transplant outcomes [6], but this assessment can be imprecise, particularly related to pre-transplant malnutrition, which is known to be common among people with advanced CF lung disease. Body mass index (BMI) is often used as an indicator of nutritional status in the pre-transplant population, but its relationship to post-transplant outcomes is inconsistent, particularly in patients who are underweight [7–15].

A potential explanation for inconsistencies in the relationship between BMI and clinical outcomes following lung transplantation is the inability of BMI to discriminate between fat mass and muscle mass. Loss of skeletal muscle mass and function, also referred to as sarcopenia, is a key component of frailty, a syndrome characterized by the accumulation of physiologic deficits that increase vulnerability to adverse events [16]. Sarcopenia is common and has been linked to mortality in advanced lung disease [17], along with worse outcomes following lung transplantation [18, 19]; however, these studies include limited numbers of patients with CF, leaving much to be understood about sarcopenia and lung transplantation in this patient population.

There are many potential mechanisms that could contribute to sarcopenia in CF including physical inactivity, inflammation, malnutrition, and cystic fibrosis transmembrane conductance regulator (CFTR) specific muscle dysfunction [20–22]. With the advent of CFTR modulator therapies patients have experienced significant improvements in weight and BMI [23]; however, the impact on lean body mass is unknown and the study of muscle mass in CF remains relevant to current clinical care. Including a measure of muscle mass in the assessment of nutritional status may provide a clearer picture of a patient’s pre-transplant risk than BMI alone. Computed tomography (CT) body composition analysis is an alternative approach that has been utilized among patients undergoing liver transplantation [24–28], but there is insufficient evidence to inform risk assessments of patients with CF who are preparing for lung transplantation [18, 19, 29].

Our primary objective was to determine whether muscle mass measured by CT is associated with post-transplant outcomes after lung transplantation for CF. Our primary outcome was survival, with secondary outcomes of days to post-transplant extubation and post-transplant hospital and intensive care unit (ICU) length of stay. Additional aims included an evaluation of the association between muscle mass measured by CT and markers of functional reserve among patients with CF, including pre-transplant 6-min walk distance and forced expiratory volume in 1 s (FEV1) % predicted. We hypothesized that lower muscle mass would be associated with poor pre-transplant functional reserve and higher mortality following lung transplantation.

## Methods

### Study design and participants

Our transplant program’s candidate selection process has evolved over the past 25 years, and like the greater lung transplant community, we have gradually shifted towards transplanting slightly sicker and older patients. Despite this gradual evolution, there were no major changes to our official listing criteria over the study period. Between May 1993 and December 2018, 152 adult CF patients received lung transplants at the University of Washington Medical Center (UWMC). Of these patients, 93 had accessible CT scans of the chest completed within one year prior to transplant. From this group we excluded individuals who underwent multi-organ transplant (e.g., heart and lung) (*n* = 2) or were re-transplant patients (*n* = 2) and those for whom no post-transplant follow-up records were available (*n* = 2). Of the remaining patients with accessible pre-transplant CT scans, 4 scans were unusable because of anatomical distortion (e.g., subcutaneous emphysema), leaving 83 patients for our total sample. This project was approved by the institutional review board at the University of Washington (STUDY00012301).

### Measures

CT scans were available in a centralized, secure picture archiving and communication system (PACS) at UWMC. For our predictor of interest, we used muscle mass measured by CT scan of the chest obtained closest in time to transplant. Using a standardized image selection protocol, we selected a single axial slice nearest the inferior aspect of the T12 vertebral body. We selected the inferior aspect to obtain measurements as close as possible to the region associated with peak skeletal muscle area [30]. The direct measure was skeletal muscle cross sectional area (cm^2^) at the T12 vertebrae, adjusted for patient stature by dividing by height in m^2^, rendering a thoracic skeletal muscle index (SMI) (cm^2^/m^2^) [30]. Tissue cross-sectional area (cm^2^) in slices was computed automatically by summing appropriate pixels using the CT Hounsfield unit (HU) range − 29 HU to 150 HU for skeletal muscle. The following muscles in the axial slice were included in our measurements: rectus abdominus, external and internal intercostals, external obliques and internal obliques (if present), latissimus dorsi, erector spinae (iliocostalis thoracis, longissimus dorsi, spinalis), and transversospinalis (if present). We did not incorporate the diaphragm or transverse abdominis in our measurements. The diaphragm, when present in T12 slices, is difficult to differentiate from adjacent solid organs. To maintain consistency the transverse abdominis was excluded as well, as it may interdigitate with the diaphragm in the T12 area, making it difficult to differentiate between these muscles. All scans were analyzed using Slice-O-Matic software (Tomovision, Magog, QC, Canada). One reviewer (ALJ) independently assessed muscle mass on all scans, and a second reviewer (KJR) assessed a random selection of 10% of scans for assessment of inter-rater reliability.

Chart abstraction was used to obtain demographics including CF genotype (F508del homozygous vs not), and additional pre-transplant patient characteristics: body mass index (BMI), positive respiratory cultures in the year preceding transplant, pretransplant lung allocation score, CF-related diabetes requiring insulin, supplemental oxygen use at rest, forced expiratory volume in 1 s (FEV1) % predicted, 6-min walk distance (6MWD), receipt of mechanical ventilation immediately prior to transplant, and use of extracorporeal life support immediately prior to transplantation. For pre-transplant BMI, pre-transplant lung allocation score, 6-min walk distance, and FEV1% predicted, we used values obtained closest in time to the CT scan used for muscle mass measurement. Post-transplant outcomes included days to extubation, hospital and ICU length of stay, and survival status (last updated January 2022). Days to extubation included days on mechanical ventilation from transplant to initial extubation. For patients discharged to acute care who required ICU readmission, ICU length of stay was calculated using the discharge date from the patient’s second admission.

### Analysis

Our assessment of interrater reliability between reviewers was measured with the intraclass correlation coefficient for a two-way mixed effects model (patients as random effect and raters as fixed effect [same two raters for all patients]) in which absolute agreement was assessed.

We evaluated the association between thoracic SMI and post-transplant survival, using multivariable Cox proportional hazards regression with robust standard errors. Due to our small sample size and limited number of deaths, we were required to be parsimonious in our inclusion of a priori potential confounders and included sex (to account for differences in muscle mass across sexes and potential relationships between sex and post-transplant outcomes) and calendar year of transplant (to account for changes over time in nutritional management in CF and transplant care) in our main model. We used sensitivity analyses to evaluate additional confounders, including CF genotype or BMI, in separate Cox proportional hazards regression models. Multivariable linear regression was used to evaluate associations between thoracic SMI and our secondary outcomes, including days to extubation and hospital and ICU length of stay. These models were adjusted for sex, calendar year of transplant, CF genotype, and BMI. Associations of pre-transplant FEV1% predicted and 6-min walk distance with thoracic SMI were examined using separate multivariable linear regression with robust standard errors, adjusting for sex and calendar year of transplant.

Additional analyses included: 1) assessment of the hazard for death by thoracic SMI using bivariate Cox proportional hazard regression stratified by sex; 2) an assessment of the probability for survival based on being above or below the median SMI, using Cox proportional hazard regression adjusted for sex; 3) an assessment of the correlation between thoracic SMI and BMI; and 4) an assessment of sex-specific median thoracic skeletal muscle index by year of transplant All Cox models were evaluated for violation of the proportional-hazards assumption, based on Schoenfeld residuals. Two-sided *p* values of ≤ 0.05 were deemed significant. Analyses were done with IBM SPSS Statistics (Version 27) and Stata/IC (Version 16.1).

## Results

Our final cohort included 83 adults with CF. For the 83 patients included in our analyses, median survival time was 6.17 years. At the time of the last survival status update, 45 were survivors (54%) and 38 were decedents (46%). The sample included similar proportions of male and female patients and had a median age at transplant of 29 years (IQR 24, 34). The median elapsed time from pre-transplant CT to transplant was 122 days (IQR 57, 188). Median skeletal muscle cross sectional area at T12 was 68.53 cm^2^ (IQR 59.02, 86.27), and median thoracic SMI was 25.62 cm^2^/m^2^ (IQR 22.10, 29.03). Interrater reliability for review of muscle mass measurements was excellent, with an intraclass correlation of 0.95 for skeletal muscle cross sectional area. Comparing men to woman, median thoracic SMI was 26.95 cm^2^/m^2^ (IQR 23.97, 31.32) for men and 22.83 cm^2^/m^2^ (IQR 21.27, 26.92) for women. Although values depend on a variety of measurement-specific factors, for healthy individuals *mean* SMI at this vertebral level has been reported as 44.1 (± standard deviation 7.7) for men and 34.0 (± standard deviation 6.6) for women [30]. Sarcopenia cutoffs at T12, using sex-specific cutoffs for ‘low’ values set at two standard deviations below the *mean* of healthy adults, have been reported as 20.8 cm^2^/m^2^ for women and 28.8 cm^2^/m^2^ for men [30]. Sex-specific median SMI by year is detailed in our supplementary material (Table E1, Figure E1). Median pre-transplant BMI was 20 kg/m^2^ (IQR 18.44, 21.44), and the Pearson correlation between thoracic SMI and BMI was 0.61 (*p* < 0.001). A complete description of patient characteristics by survival status is included in Table 1, and information about positive respiratory cultures in the year preceding transplant is available in online supplement Table E2 .

**Table 1:** Patient characteristics by survival status^a^

Characteristic	Survivors		Decedents		Total	
	n	Descriptive^a^	n	Descriptive^a^	n	Descriptive^a^
Sex	45		38		83	
Male		15 (33.3)		25 (65.8)		40 (48.2)
Female		30 (66.7)		13 (34.2)		43 (51.8)
Age at transplant, median (IQR)	45	31 (25, 38.5)	38	27.5 (23.75, 32.25)	83	29 (24, 34)
Transplant year, median (range)	45	2013 (2001, 2018)	38	2010 (2003, 2017)	83	2011 (2001, 2018)
Genotype: F508del homozygote	42		31		73	
No		18 (42.9)		8 (25.8)		26 (35.6)
Yes		24 (57.1)		23 (74.2)		47 (64.4)
Pre-transplant FEV1% predicted, median (IQR)^b^	45	22 (18.5, 26)	38	21 (18, 27.25)	83	22 (18, 26)
Pre-transplant 6MWD in feet, median (IQR)^b^	44	1082.5 (780, 1333)	35	1070 (857, 1205)	79	1070 (826, 1275)
Pre-transplant lung allocation score, median (IQR)^b^	36	41.0 (36.2, 43.1))	22	37.3 (35.0, 42.0)	58	39.6 (36.0, 43.0)
CF-related diabetes requiring insulin	45		38		83	
No		20 (44.4)		22 (57.9)		42 (50.6)
Yes		25 (55.6)		16 (42.1)		41 (49.4)
Pre-transplant oxygen at rest	45		37		82	
No		15 (33.3)		7 (18.9)		22 (26.8)
Yes		30 (66.7)		30 (81.1)		60 (73.2)
Pre-transplant mechanical ventilation^c^	45		38		83	
No		41 (91.1)		35 (92.1)		76 (91.6)
Yes		4 (8.9)		3 (7.9)		7 (8.4)
Pre-transplant extracorporeal life support^c^	45		38		83	
No		43 (95.6)		36 (94.7)		79 (95.2)
Yes		2 (4.4)		2 (5.3)		4 (4.8)
Pretransplant body dimensions, median (IQR)	45		38		83	
Height, meters^b^		1.66 (1.58, 1.73)		1.675 (1.61, 1.73)		1.67 (1.60, 1.73)
Skeletal muscle cross sectional area at T12, cm^2^		66.94 (56.78, 85.44)		70.74 (66.07, 89.46)		68.53 (59.02, 86.27)
Thoracic skeletal muscle index, cm^2^/m^2^		24.17 (21.51, 28.57)		26.01 (22.75, 29.36)		25.62 (22.10, 29.03)
Weight in kilograms^b^		55.60 (50.65, 61.65)		53.85 (49, 63.5)		55.20 (50.40, 62.00)
Body mass index^b^		20.03 (18.44, 21.78)		20.10 (18.51, 21.27)		20.03 (18.44, 21.44)

a Unless otherwise specified, each cell contains the number of cases and percentage of valid cases for the column

b Values obtained closest in time to the pre-transplant CT scan used for muscle mass measurement

c Used immediately prior to transplantation

**Fig. 1:**
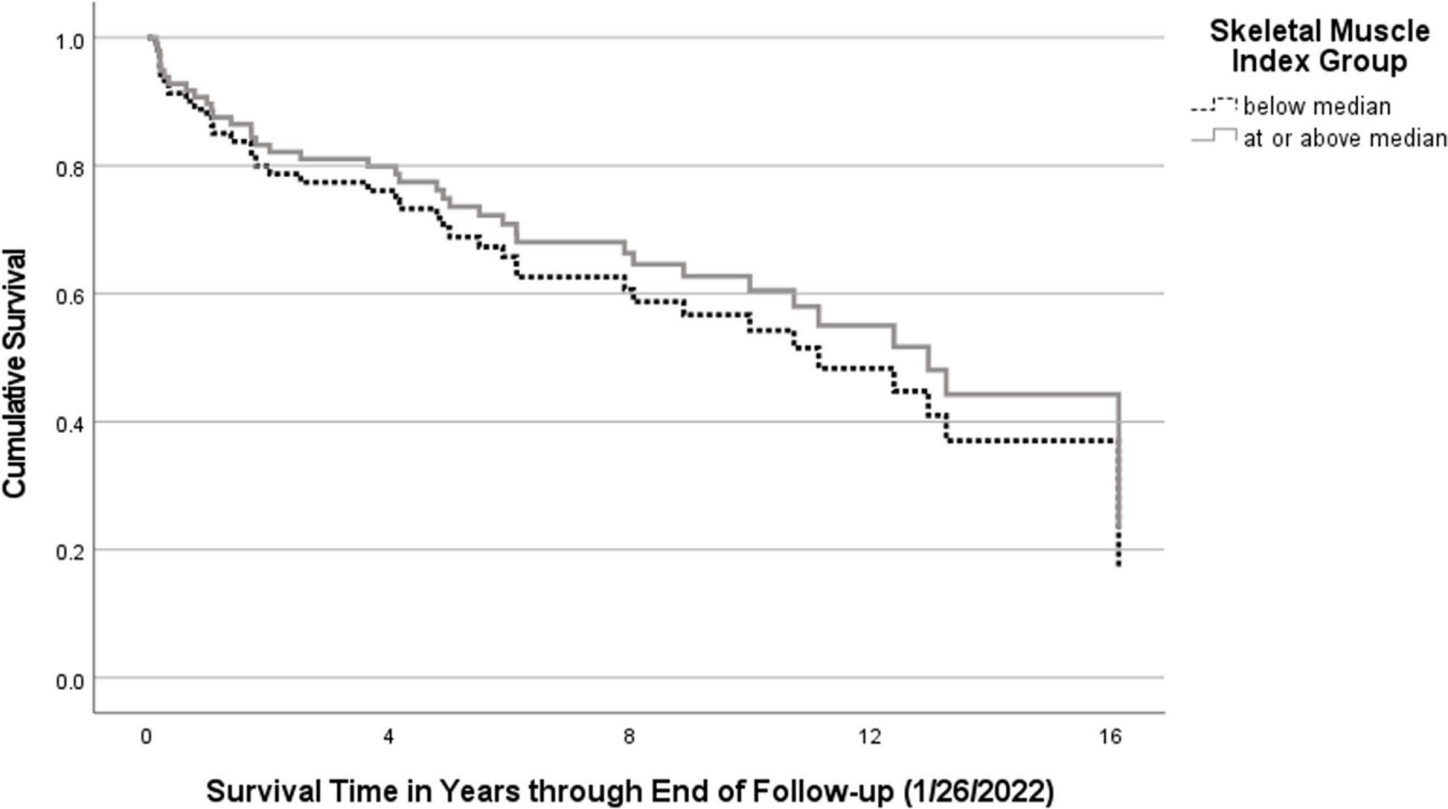
Post-transplant survival probability by SMI group, based on Cox proportional hazards regression model, adjusted for sex (two groups plotted at the mean value for sex)

**Table 2:** Hazard for post-transplant death^a^

Predictors	HR	*p*	95% CI
Thoracic skeletal muscle index	1.03	0.53	0.95, 1.11
Female	0.53	0.09	0.25, 1.09
Transplant year	1.00	0.93	0.92, 1.09

a Sample size = 83. Results are based on a multi-predictor Cox proportional hazards regression model with robust standard errors. Test of proportional hazards assumption: χ^2^ = 1.18, 3 df, *p* = 0.757. Thirty-eight deaths; 45 patients censored at end of follow-up on 1/26/22

### Thoracic skeletal muscle index and post-transplant outcomes

Using multivariable Cox proportional hazards regression, we found no significant association between thoracic SMI and death adjusting for sex and calendar year of transplant (HR = 1.03; 95% CI 0.95, 1.11) (Table 2). Additional analyses adjusting for CF genotype and BMI in separate models also demonstrated a non-significant relationship between thoracic SMI and death (online supplement Tables E3-E4). A sensitivity analysis assessing the bivariate association between thoracic SMI and death, stratified by sex, similarly showed no significant association (males HR 1.03; 95% CI 0.93, 1.14 and females HR 1.00; 95% CI 0.90, 1.11). Nor were there differences in survival between two groups divided at the median SMI, using Cox regression adjusted for sex, although the 95% CI was large (HR 1.22; 95% CI 0.61, 2.43) (Fig. 1). Multivariable linear regression models examining the relationship between thoracic SMI and days to extubation, and hospital and ICU length of stay showed no significant associations. Complete results with covariate adjustments are included in online supplement Tables E5-E7.

### Thoracic skeletal muscle index and markers of pre-transplant functional reserve

We found a significant association between thoracic SMI and pre-transplant FEV1% predicted, where higher SMI was associated with higher FEV1% predicted (b 0.39; *p* = 0.002; 95% CI 0.14, 0.63) (Table 3). The association between thoracic SMI and pre-transplant 6MWD was not statistically significant (online supplement Table E8).

**Table 3:** Association between thoracic SMI and pre-transplant FEV1% predicted^a^

Predictors	b	*p*	95% CI
Thoracic skeletal muscle index	0.39	**0.002**	0.14, 0.63
Female	1.63	0.23	-1.03, 4.28
Transplant year	-0.04	0.81	-0.34, 0.27

a. Sample size = 83. Results based on a multi-predictor linear regression model with robust standard errors

## Discussion

In this study we were able to successfully quantify skeletal muscle mass in a cohort of patients with advanced CF lung disease who underwent lung transplantation. Measurements had very high interrater reliability, and compared to population norms, both men and women in our population had low SMI values [30]. However, it is unclear if cutoffs specified for healthy adults can be applied to patients with chronic illness or used to define sarcopenia in CF [30]. We did not identify an association between pre-transplant thoracic SMI and death following lung transplantation, nor did we identify associations between pre-transplant thoracic SMI and days to post-transplant extubation or hospital or ICU length of stay. Given the 95% CI we observed, our primary and sensitivity analyses ruled out a large association. We did identify an association between pre-transplant thoracic SMI and pre-transplant FEV1% predicted, a marker of lung disease severity.

The evaluation for lung transplantation involves assessing a patient’s indication for transplant and identifying potential factors that could increase the risk of transplant [31]. The most recent guidelines on selection of lung transplantation candidates recommend using BMI as a component of the selection process [6], and BMI is the only variable in the lung allocation score addressing a patient’s body composition [32]. However, there is debate about the importance of BMI in the selection process, with new evidence challenging existing notions about the relationship between BMI and post-transplant outcomes particularly as it relates to underweight CF patients [33, 34]. Because BMI does not discriminate between fat mass and fat-free body mass, it is not an ideal measure of body composition.

The presence of low muscle mass, or sarcopenia, may be a better indicator of nutritional health. Low muscle mass has been identified in normal, overweight, and obese adults [35–38], underscoring its potential to outperform BMI as a measure of body composition. Sarcopenia is also closely linked to the concept of frailty [39–41]. Frailty has been associated with morbidity and mortality in kidney [42–44] and liver transplant [45] populations, but less is known about frailty in lung transplantation [18]. Existing data suggest that frailty is common in lung transplant candidates and associated with a higher rate of delisting or death before lung transplant [46]. Likewise, sarcopenia has been associated with worse post-transplant outcomes in other solid organ transplant populations [25–28, 47], but few studies have addressed sarcopenia in lung transplant recipients [18, 19, 29].

It is important to note that existing studies include very few patients with CF [18, 19, 29], a population where mechanisms for muscle loss may be quite different compared to patients with other forms of advanced lung disease, like chronic obstructive pulmonary disease (COPD) or idiopathic pulmonary fibrosis (IPF). Differences tied to the etiology of advanced lung disease may provide a potential explanation for our results, which suggest the absence of a significant relationship between thoracic SMI and post-transplant survival in patients with CF. Both IPF and COPD occur later in life and result in transplantation at an older age. It may be that, compared to patients with other forms of advanced lung disease, the younger age of patients with CF offers a greater level of reserve that allows one to tolerate lower pre-transplant muscle mass. Younger patients have also not accrued the cumulative years of chronic illness born by those with COPD or IPF, particularly cardiovascular disease. Cardiovascular disease is extremely rare in CF thus potentially protecting this patient group in the post-transplant period. Also, despite a low muscle mass prior to transplantation, the ability to rehabilitate after transplant may be more robust among younger patients. Future research is needed to understand the relationship between muscle mass and long-term post-transplant outcomes like FEV1 and 6WMD, as well as patient-reported outcomes related to quality of life.

Although there was no association between thoracic SMI and our post-transplant outcomes, the identified relationship between pre-transplant thoracic SMI and pre-transplant FEV1% predicted suggests the potential role of muscle mass measurement as an additional indicator of severity of illness in CF. Given our sample size, we were unable to assess whether there was an interaction between SMI and FEV1% predicted on our primary outcome. Additionally, for patients who are normal weight or overweight, the presence of sarcopenia may prompt nutritional interventions for severity of illness which may not have been indicated based on BMI alone. We found a significant correlation between BMI and thoracic SMI; however, the strength of correlation (0.61) suggests that both measurements may provide important information, independently of one another. Moreover, in a patient population where a narrow range of BMI is expected, other measures may be necessary to develop an accurate impression of body composition. Additional research is needed to further elucidate the relationship between muscle mass, measurements of functional reserve, and BMI to better inform our understanding of frailty in patients with CF.

Our study has several important limitations. First, although our cohort included a relatively large number of patients transplanted for CF, the use of data from a single center limits generalizability, and our sample size may have affected our ability to detect associations. Of the 59 patients transplanted at our center during the study period who were not included, 35 had no available CT scans and 24 had scans done more than one year prior to transplant. Among the 35 patients without available scans, 89% were transplanted in the pre-lung allocation score era (i.e., prior to 2005). The ability to include only those with available imaging, within our specified time frame, may have contributed to some degree of selection bias for the following reasons: changes in clinical practice and the health of transplant recipients changed over time, including not only CFTR modulators but also those related to early diagnosis via newborn screening, nutritional supplementation, and consistent implementation of a CF care model that have improved clinical outcomes; and individuals without available scans in the year preceding transplant may have differed from those who did have available scans. We did adjust for calendar year of transplant in our analyses, to account for changes over time in CF management and transplant care, and we felt it important to maintain the 1-year time frame for scan inclusion, given potential changes in muscle mass over time. Second, the techniques used to measure muscle mass in patients with pulmonary disease are highly variable [19, 29, 48–50]. It is possible that the methods used in other studies provide a better representation of total body muscle mass than measurements performed at T12. Muscle mass measured at the 3^rd^ lumbar vertebrae (L3) by CT is considered the reference standard for estimating total body skeletal muscle mass [51]. However, CT scans of the chest do not include L3, necessitating measurement elsewhere. Evidence suggests T12 is an acceptable position, and based on its presence on CT scans of the chest and its correlation with muscle area measured in the lumbar region, it was the most acceptable choice for our purposes [52]. Third, because our study was limited to patients who underwent lung transplantation, our cohort may have been a “healthier” or less frail population than if it had included all candidates with CF who were evaluated for transplantation. The number of candidates with CF at our institution who were declined for transplant or who died on the waiting list was quite small during the study period, therefore precluding this analysis, but it should be noted that the relationship between thoracic SMI and clinical outcomes may be different in those who are deemed to be poor candidates for transplant by clinical teams. Fourth, for our outcome of days to extubation, we included days on mechanical ventilation from transplant to initial extubation and thus did not account for instances of reintubation. Lastly, our study includes patients cared for before highly effective CFTR modulator therapies became available for most of the CF population. While existing data suggests improvements in BMI with these therapies [23], and we expect improvements in BMI to correlate with increases in muscle mass, sarcopenia in CF remains relevant until this relationship is better understood.

In summary, despite a growing body of evidence highlighting sarcopenia as a potentially valuable measure for transplant assessments, we did not find evidence of a relationship between thoracic SMI and post-transplant outcomes for people with CF. We did, however, find a relationship between muscle measurements and pre-transplant pulmonary function, which confirms the potential value of examining sarcopenia as a marker of disease severity in CF. Additional research is necessary to understand the prognostic potential of sarcopenia for patients with CF approaching lung transplantation. Finally, to advance our understanding of sarcopenia in patients with CF, uniformity in the methods used to obtain muscle mass measurement will be essential, along with concerted efforts to establish a consistent approach to defining sarcopenia in patients with advanced lung disease [53].

## Supplementary Information

**Additional file 1:** **Table E1.** Sex-specific median thoracic SMI by year of transplant. **Figure E1.** Sex-specific median thoracic SMI by year of transplant. **Table E2.** Positive respiratory cultures in the year preceding transplant by survival status. **Table E3.** Hazard for post-transplant death, addition of genotype as a potential confounder^a^. **Table E4.** Hazard for post-transplant death, addition of BMI as a potential confounder^a^. **Table E5.** Days from Transplant to First Extubation^a^. **Table E6.** Days from Transplant to Hospital Discharge^a^. **Table E7.** Days from Transplant to ICU Dischargea. **Table E8.** Pre-transplant 6MWD^a^.

## Data Availability

The datasets used and/or analyzed during the current study are available from the corresponding author on reasonable request.

## Abbreviation

6MWD: 6-Minute walk distance
BMI: Body mass index
CF: Cystic fibrosis
CFTR: Cystic fibrosis transmembrane conductance regulator
COPD: Chronic obstructive pulmonary disease
CT: Computed tomography
FEV1: Forced expiratory volume in 1 second
HU: Hounsfield unit
ICU: Intensive care unit
IPF: Idiopathic pulmonary fibrosis
PACS: Picture archiving and communication system
SMI: Skeletal muscle index
UWMC: University of Washington medical center
