# Supplementary material for: Computed tomography body composition and clinical outcomes following lung transplantation in cystic fibrosis
Source: BMC Pulm Med. 2023 Mar 30;23:105. doi: 10.1186/s12890-023-02398-4 (PMC10062009; doi:10.1186/s12890-023-02398-4)
Supplement: Supplementary file 1 — Additional file 1: Table E1. Sex-specific median thoracic SMI by year of transplant. Figure E1. Sex-specific median thoracic SMI by year of transplant. Table E2. Positive respiratory cultures in the year preceding transplant by survival status. Table E3. Hazard for post-transplant death, addition of genotype as a potential confoundera. Table E4. Hazard for post-transplant death, addition of BMI as a potential confoundera. Table E5. Days from Transplant to First Extubationa. Table E6. Days from Transplant to Hospital Dischargea. Table E7. Days from Transplant to ICU Dischargea. Table E8. Pre-transplant 6MWDa. [file 12890_2023_2398_MOESM1_ESM.docx]

**Online Supplement:** Computed tomography body composition and clinical outcomes following lung transplantation in cystic fibrosis

| **Table E1. Sex-specific median thoracic SMI by year of transplant** | | | | |
| --- | --- | --- | --- | --- |
|  | Male | | Female | |
| Year | n | median | n | median |
| 2001 | 0 |  | 1 | 20.004009 |
| 2002 | 0 |  | 0 |  |
| 2003 | 1 | 37.539990 | 0 |  |
| 2004 | 2 | 26.453542 | 0 |  |
| 2005 | 3 | 28.495133 | 1 | 22.101902 |
| 2006 | 3 | 23.401384 | 0 |  |
| 2007 | 3 | 26.072664 | 2 | 20.695972 |
| 2008 | 4 | 26.249082 | 2 | 24.441875 |
| 2009 | 1 | 27.555880 | 5 | 28.937927 |
| 2010 | 2 | 26.278847 | 1 | 26.916998 |
| 2011 | 7 | 26.518536 | 4 | 24.810487 |
| 2012 | 3 | 23.897507 | 3 | 22.945041 |
| 2013 | 2 | 32.180928 | 2 | 22.169812 |
| 2014 | 4 | 32.915223 | 4 | 23.180340 |
| 2015 | 2 | 22.153565 | 6 | 22.142874 |
| 2016 | 1 | 27.203265 | 3 | 30.325069 |
| 2017 | 1 | 34.676754 | 4 | 20.833938 |
| 2018 | 1 | 29.027269 | 5 | 23.013528 |
| TOTAL | 40 | 26.947954 | 43 | 22.828512 |
| Dif in md by year, *p*^a^ |  | 0.490 |  | 0.124 |
| 1. Performed using the median test in SPSS. | | | | |

**Figure E1. Sex-specific median thoracic SMI by year of transplant**

| **Table E2. Positive respiratory cultures in the year preceding transplant by survival status** | | | | | | |
| --- | --- | --- | --- | --- | --- | --- |
| Characteristic | Survivors | | Decedents | | Total | |
|  | n | n (%) | n | n (%) | n | n (%) |
| Pseudomonas | 45 |  | 38 |  | 83 |  |
| No |  | 5 (11.1) |  | 7 (18.4) |  | 12 (14.5 |
| Yes |  | 40 (88.9) |  | 31 (81.6) |  | 71 (85.5) |
| Methicillin-sensitive staphylococcus aureus | 45 |  | 38 |  | 83 |  |
| No |  | 29 (64.4) |  | 25 (65.8) |  | 54 (65.1) |
| Yes |  | 16 (35.6) |  | 13 (34.2) |  | 29 (34.9) |
| Methicillin-resistant staphylococcus aureus | 45 |  | 38 |  | 83 |  |
| No |  | 31 (68.9) |  | 30 (78.9) |  | 61 73.5) |
| Yes |  | 14 (31.1) |  | 8 (21.1) |  | 22 (26.5) |
| Stenotrophomonas | 45 |  | 38 |  | 83 |  |
| No |  | 34 (75.6) |  | 33 (86.8) |  | 67 (80.7) |
| Yes |  | 11 (24.4) |  | 5 (13.2) |  | 16 (19.3) |
| Burkholderia cepacia complex | 45 |  | 38 |  | 83 |  |
| No |  | 41 (91.1) |  | 35 (92.1) |  | 76 (91.6) |
| Yes |  | 4 ( 8.9) |  | 3 ( 7.9) |  | 7 ( 8.4) |
| Achromobacter | 45 |  | 38 |  | 83 |  |
| No |  | 34 (75.6) |  | 31 (81.6) |  | 65 (78.3) |
| Yes |  | 11 (24.4) |  | 7 (18.4) |  | 18 (21.7) |
| Aspergillus | 45 |  | 38 |  | 83 |  |
| No |  | 23 (51.1) |  | 22 (57.9) |  | 45 (54.2) |
| Yes |  | 22 (48.9) |  | 16 (42.1) |  | 38 (45.8) |
| Non-fumigatus mold | 45 |  | 38 |  | 83 |  |
| No |  | 41 (91.1) |  | 36 (94.7) |  | 77 (92.8) |
| Yes |  | 4 ( 8.9) |  | 2 ( 5.3) |  | 6 ( 7.2) |
| Non-tuberculous mycobacteria | 45 |  | 38 |  | 83 |  |
| No |  | 42 (93.3) |  | 32 (84.2) |  | 74 (89.2) |
| Yes |  | 3 ( 6.7) |  | 6 (15.8) |  | 9 (10.8) |
| M. abscessus | 45 |  | 38 |  | 83 |  |
| No |  | 45 (100.0) |  | 37 (97.4) |  | 82 (98.8) |
| Yes |  | 0 ( 0.0) |  | 1 ( 2.6) |  | 1 ( 1.2) |

| **Table E3. Hazard for post-transplant death, addition of genotype as a potential confounder**^a^ | | | |
| --- | --- | --- | --- |
| Predictors | HR | *p* | 95% CI |
| Thoracic skeletal muscle index | 1.07 | 0.12 | 0.98, 1.16 |
| Female | 0.69 | 0.38 | 0.30, 1.58 |
| Transplant year | 1.03 | 0.55 | 0.94, 1.13 |
| Genotype: F508del homozygote | 1.20 | 0.69 | 0.48, 3.00 |
| a. Sample size = 73. Results are based on a multi-predictor Cox proportional hazards regression model with robust standard errors. Test of proportional hazards assumption: χ^2^ = 1.25, 4 df, *p*=0.870. Thirty-one deaths; 42 patients censored at end of follow-up on 1/26/22. | | | |

| **Table E4. Hazard for post-transplant death, addition of BMI as a potential confounder**^a^ | | | |
| --- | --- | --- | --- |
| Predictors | HR | *p* | 95% CI |
| Thoracic skeletal muscle index | 1.04 | 0.40 | 0.95, 1.15 |
| Female | 0.54 | 0.10 | 0.26, 1.12 |
| Transplant year | 1.00 | 0.95 | 0.92, 1.09 |
| Body mass index | 0.96 | 0.59 | 0.81, 1.12 |
| a. Sample size = 83. Results are based on a multi-predictor Cox proportional hazards regression model with robust standard errors. Test of proportional hazards assumption: χ^2^ = 1.51, 4 df, *p*=0.824. Thirty-eight deaths; 45 patients censored at end of follow-up on 1/26/22. | | | |

| **Table E5. Days from Transplant to First Extubation**^a^ | | | |
| --- | --- | --- | --- |
| Predictors | b | *p* | 95% CI |
| Thoracic skeletal muscle index | 0.28 | 0.26 | -0.21, 0.77 |
| Female | 0.71 | 0.45 | -1.16, 2.58 |
| Transplant year | 0.22 | 0.23 | -0.14, 0.58 |
| Genotype: F508del homozygote | 0.14 | 0.91 | -2.29, 2.57 |
| Body mass index | 0.51 | 0.35 | -0.57, 1.60 |
| a. Sample size = 73. Results are based on a multi-predictor linear regression model with robust standard errors. | | | |

| **Table E6. Days from Transplant to Hospital Discharge**^a^ | | | |
| --- | --- | --- | --- |
| Predictors | b | *p* | 95% CI |
| Thoracic skeletal muscle index | -0.72 | 0.24 | -1.92, 0.49 |
| Female | 1.46 | 0.71 | -6.31, 9.23 |
| Transplant year | 1.16 | 0.06 | -0.05, 2.37 |
| Genotype: F508del homozygote | -6.78 | 0.22 | -17.69, 4.13 |
| Body mass index | 2.16 | 0.11 | -0.50, 4.81 |
| a. Sample size = 73. Results are based on a multi-predictor linear regression model with robust standard errors. Two patients were discharged from the hospital at death. | | | |

| **Table E7. Days from Transplant to ICU Discharge**^a^ | | | |
| --- | --- | --- | --- |
| Predictors | b | *p* | 95% CI |
| Thoracic skeletal muscle index | 0.06 | 0.88 | -0.78, 0.90 |
| Female | 3.78 | 0.10 | -0.81, 8.37 |
| Transplant year | 0.72 | 0.15 | -0.27, 1.70 |
| Genotype: F508del homozygote | -2.69 | 0.52 | -10.98, 5.60 |
| Body mass index | 0.95 | 0.34 | -1.03, 2.94 |
| a. Sample size = 73. Results are based on a multi-predictor linear regression model with robust standard errors. | | | |

| **Table E8. Pre-transplant 6MWD**^a^ | | | |
| --- | --- | --- | --- |
| Predictors | b | *p* | 95% CI |
| Thoracic skeletal muscle index | -3.71 | 0.67 | - 21.10, 13.68 |
| Female | -170.99 | 0.06 | -350.90, 8.93 |
| Transplant year | -6.50 | 0.52 | - 26.60, 13.60 |
| 1. Sample size = 79. Results based on a multi-predictor linear regression model with robust standard errors. | | | |
